# Commentary: Factors Associated With Mortality Among the COVID-19 Patients Treated at Gulu Regional Referral Hospital

**DOI:** 10.3389/fpubh.2022.931309

**Published:** 2022-07-05

**Authors:** Ronald Olum, Joseph Baruch Baluku

**Affiliations:** ^1^Department of Internal Medicine, St. Francis Hospital Nsambya, Kampala, Uganda; ^2^Makerere University Lung Institute, Makerere University, Kampala, Uganda; ^3^Division of Pulmonology, Kiruddu National Referral Hospital, Kampala, Uganda

**Keywords:** COVID, mortality, Uganda, outcome, commentary

## Introduction

We read with interest the recently published article by Baguma and colleagues in the journal (1), reporting mortality rate among patients with COVID-19 admitted at Gulu Regional Referral Hospital, Gulu, Uganda. This retrospective chart review further attempted to describe factors associated with COVID-19 mortality at this tertiary hospital. Overall, of the 664 participants included in the analysis; mortality rate was 4.8% (*n* = 32). The authors indicate in the results and discussion sections that female gender, participants aged 50 years or older, diabetes mellitus and other chronic conditions were associated with higher odds of mortality. Furthermore, the study by Baguma et al. (1) highlights that COVID-19 patients with certain symptoms at presentation such as tiredness, general aches and pains, loss of speech and movement, had higher odds of mortality. The paper further emphasizes through additional analysis that the increased odds of mortality among the female patients could be significantly attributed to chronic obstructive pulmonary disease and cardiovascular diseases.

Whereas, this study adds to the literature on COVID-19 outcomes in Uganda and Africa at large, we have concerns on application of statistics, data presentation, interpretation of the findings and conclusions derived from the study. First, the study does not present the characteristics of all the 664 patients included in the analysis. As such we are unable to have a picture of the distribution of demographics of all the patients admitted at Gulu Regional Referral Hospital. This is consistently seen in all the tables in the article, describing only data on the 32 patients who died. As such, it is impossible to visually compare outcomes among those who died and those lived. Secondly, the low mortality rate provides an important statistical limitation in making conclusions regarding factors associated with mortality. This is further evidenced by the wide confidence intervals across all the significant variables, suggesting poor precision. This important limitation was not acknowledged in the paper.

Regarding interpretation, we are puzzled with how the authors interpreted the outputs of the regression analysis. For example, the authors emphasized throughout the article that female gender was associated with higher odds of mortality. However, the adjusted odds ratio is 0.220 (95% CI: 0.059–0.827), suggesting a lower likelihood of mortality instead. In other words, male participants had 4.5-fold higher odds of dying compared to their female counterparts. This interpretation also applies for symptoms of tiredness (AOR: 0.059, 95% CI: 0.009–0.371), general body aches and pains (AOR: 0.066, 95% CI: 0.007–0.605), and loss of speech and movements (AOR: 0.134, 95% CI: 0.270–0.660). Providing the reference groups for which these outcomes are being compared to will give a better perspective. In Table 2, the description of the bivariate analysis without a control group (died vs. alive) is questionable. Moreover, in the main text, the authors use chi-square (X^2^) value as a measure of the strength of associations. The title of Table 4 talks about “factors associated with female gender” and still includes female gender among the variables. This title is likely to be incorrect.

## Discussion

### COVID-19 Mortality in Uganda

The COVID-19 pandemic in Uganda, just like many African countries have been described as “mild” compared to other high-income countries. To date, about 166,996 cases have been confirmed, accounting for <1% of the overall population (2). The mortality rate in Uganda has generally been low, at 2.2% as of April 11, 2022. The mortality rates have also differed significantly by the COVID-19 waves Uganda has experienced. To date, Uganda has experienced three waves of COVID-19; the first wave was in mid-late 2020, second wave in April 2021–August 2021, and the third wave in December 2021–February 2022. Each of these waves were characterized by predominantly different SARS-COVID-2 variants: alpha variant in the first, delta in the second, and omicron in the third wave. The mortality rates have also been different in all the three waves with the highest being in delta, followed by omicron and alpha variants.

It is important to note that the study by Baguma et al. did not compare their study findings to similar cohorts in Uganda, as shown in previously published studies. We reported no mortality during the first 100 cases of COVID-19 in Uganda (March–May 2020) (3), in agreement with findings by Kirenga et al. for the first 56 COVID-19 patients in Uganda (4). Similarly, a study of 146 COVID-19 patients admitted at the national COVID-19 treatment center in Uganda showed no mortality (5). Conversely, our study of 477 patients admitted during the second wave of COVID-19 at Mulago National Referral Hospital, Uganda, showed a high mortality rate of 36.5% (6). We believe if the authors stratified by these epidemic phases, a more meaningful interpretation of the mortality would be made by the readers.

### Factors Associated With COVID-19 Mortality

A large systematic review (7) of 42 studies including 423,117 COVID-19 patients showed that older age, male gender and a number of comorbidities (e.g., diabetes mellitus, COPD, cardiovascular disorders, etc.) are associated with an increased risk of mortality. These have been consistent in other systematic reviews and meta-analyses as Chidambaram et al. (8) and Du et al. (9). However, Baguma et al. reported that female gender was associated with increased odds of mortality, but this is a clear misinterpretation of their statistical output. During the second wave of COVID-19 in Uganda, we showed 1.4-fold higher odds of death among females than males. Notwithstanding concerns on the interpretation of the adjusted odds ratio in the study by Baguma et al. there seems to be inconclusive findings in certain countries regarding gender and COVID-19 mortality, as reported in the systematic review by Taylor et al. (10).

In conclusion, whereas the study by Baguma et al. adds to the existing body of knowledge on COVID-19 outcomes in resource constrained settings, we suggest that care must be taken while interpreting their findings. The study suffers from misinterpretation of statistical outputs, inadequate data presentation and lack of appraisal of local literature that ought to have been addressed.

## Author Contributions

All authors listed have made a substantial, direct, and intellectual contribution to the work and approved it for publication.

## Conflict of Interest

The authors declare that the research was conducted in the absence of any commercial or financial relationships that could be construed as a potential conflict of interest.

## Publisher's Note

All claims expressed in this article are solely those of the authors and do not necessarily represent those of their affiliated organizations, or those of the publisher, the editors and the reviewers. Any product that may be evaluated in this article, or claim that may be made by its manufacturer, is not guaranteed or endorsed by the publisher.
